# The role of miR-133a in silibinin-mediated inhibition of the PI3K/AKT/mTOR pathway in MCF-7 breast carcinoma cells

**DOI:** 10.22099/MBRC.2024.48818.1903

**Published:** 2024

**Authors:** Mohammadjavad Hossein-Tehrani, Roghayeh Abbasalipourkabir, Nasrin Ziamajidi

**Affiliations:** 1 Department of Clinical Biochemistry, School of Medicine, Hamadan University of Medical Sciences, Hamadan, Iran; 2 Molecular Medicine Research Center, Hamadan University of Medical Science, Hamadan, Iran

**Keywords:** Breast neoplasm, Silybin, miR-133a, EGFR, PI3K

## Abstract

Breast cancer is particularly severe in women. Research highlights the crucial role of miRNAs in key cellular processes, showcasing their intricate interactions with the oncogenic PI3K/AKT/mTOR (PAM) signaling pathway and underscoring their significant role as tumor suppressors. The effect of silibinin on cell growth and survival was evaluated using an MTT assay. Bioinformatics analysis identified putative miR-133a targets inside the PAM pathway. After incubating MCF-7 cells with silibinin, we measured miR-133a, *EGFR*, *PI3K*, *AKT*, *PTEN*, and *mTOR* expression levels using qRT-PCR. Furthermore, protein expression levels of mTOR were assessed using Western blotting. The MTT experiment displayed that silibinin effectively inhibits MCF-7 cell proliferation in a time- and dose-dependent manner. Silibinin's IC_50_ value, determined at 370 μM after 48 hours, was established. qRT-PCR analysis at this IC_50_ concentration highlighted reduced expression of *EGFR*, *PI3K*, *AKT*, *PTEN*, and *mTOR* mRNAs, alongside increased miR-133a expression. Notably, miR-133a exhibited a negative correlation with both *EGFR* and *PIK3C2A* expression. Furthermore, western blotting confirmed silibinin's capacity to diminish p-mTOR protein levels, the ultimate element of the PAM signaling pathway. The findings enhance comprehension of silibinin's impact on PAM signaling and miR-133a expression, offering promise for targeted therapies in disrupting oncogenic pathways in MCF-7 breast cancer cells. This insight could advance breast cancer treatment strategies.

## INTRODUCTION

Breast cancer stands out as the most common form of cancer on a global scale. Each year, there are over 2.3 million reported cases of breast cancer worldwide, positioning it as the primary or secondary cause of cancer-related fatalities among women in 95% of countries [1,2]. The dysregulation of cell signaling pathways, notably the PI3K/AKT/mTOR (PAM) pathway, significantly contributes to tumor cell proliferation and therapy resistance across a range of malignancies, encompassing breast cancer as well [3]. Consequently, this scenario emphasizes the pivotal importance of PAM within the realm of breast cancer, positioning it as an indispensable therapeutic target for diagnosing and prognosing individuals grappling with this condition [3]. Despite the notable strides made in diagnosing and managing breast cancer, the ailment continues to predominantly elude a definitive cure [4]. Furthermore, treating advanced stages of cancer not only imposes substantial financial burdens on patients and society but also harbors the risk of inefficacy and grave clinical complications [5]. The pursuit of an efficient cancer treatment to improve community health presents a critical challenge in the healthcare system. This is particularly significant due to the ongoing rise in annual cancer-related mortality rates, despite the variety of treatment options. Hence, there is a strong necessity to investigate promising biomarkers and targeted therapies for cancer [6]. 

Although clinically utilized treatments like surgery, chemotherapy, and radiotherapy have significant and lasting side effects, efforts have been made to address this issue. Exploring the use of organic and herbal compounds targeting specific signaling pathways could offer advantageous alternatives [7, 8]. Silibinin, an herbal flavonoid found in milk thistle seeds (*Silybum marianum L*) of the Asteraceae family, serves as the primary component of silymarin. Present understanding suggests that silibinin shares the same biological characteristics as silymarin [9]. Silibinin exhibits diverse antitumor effects, including cell cycle arrest and the targeted inhibition of signaling pathways such as AKT and Mitogen-activated protein kinases (MAPKs). Importantly, it doesn't demonstrate significant cytotoxicity in normal cells. In the fight against breast cancer, silibinin's importance stems from its anticancer properties, though the precise molecular mechanisms driving these effects remain incompletely elucidated [10, 11].

MiRNAs, short non-coding RNAs ranging from 18 to 23 nucleotides, exert a post-transcriptional negative impact on gene regulation through their connection to the 3'-untranslated regions (3'-UTRs) of target mRNAs. This interaction can lead to degradation of mRNA or the hindrance of translation processes [12]. Bioinformatics research has demonstrated that miRNAs regulate over 30% of human protein-coding genes, highlighting their function in multiple biological procedures such as apoptosis, differentiation, and cell division. The disrupted expression of miRNAs is associated with human carcinogenesis and cancer development, emphasizing their capacity as oncogenes or tumor suppressor genes [13]. MiR-133a stands out among miRNAs as a tumor suppressor, holding significant promise as a biomarker and prognostic indicator in various cancer types, including lung, ovarian, and breast cancer. Its impact extends to gene regulation, including the inhibition of breast cancer development by targeting genes like the epidermal growth factor receptor (EGFR) [5]. Therefore, miR-133a emerges as a potential therapeutic target for breast cancer, necessitating the development of creative strategies. Combining miR-133a with traditional cancer treatments becomes crucial for effectively addressing this challenge. Regrettably, despite numerous studies investigating the influence of silibinin on the expression of diverse microRNAs within various cell signaling pathways, the specific role of miR-133a in silibinin treatment remains elusive. This study reveals compelling evidence that silibinin enhances the efficacy of miR-133a through the PAM signaling pathway in breast cancer cells.

## MATERIAL AND METHODS

**Cell Culture: **The MCF-7 cellular lineage, sourced from human breast carcinoma, was obtained from the National Cell Bank of Iran (Pasteur Institute of Iran). All cells were cultured under standard laboratory conditions at 37°C with 5% CO_2 _and 90-95% humidity. They were maintained in Dulbecco-modified Eagle medium (DMEM; Bio Idea, Iran, Tehran) supplemented with 10% fetal bovine serum (FBS; Kiazist, Iran, Tehran), and 1% combination of streptomycin and penicillin (Bio Idea, Iran, Tehran). All interventions were carried out on logarithmically expanding cells. To prevent instability-induced genetic sway, the focus of the research using the MCF-7 cell line was constrained to the first ten passages from the initial flask. The DMEM medium was replaced every three days, and the cells were passaged upon reaching a confluence of 65 to 80%. Silibinin (Sigma, USA) was dissolved in dimethyl sulfoxide (DMSO; Kiazist, Iran, Tehran) to prepare the initial solution. Complete DMEM media was used for dilution, ensuring that the concentration of DMSO never exceeded 0.1% (v/v) throughout the experiment to minimize the potential cytotoxic effects on cells.

**Figure 1:**
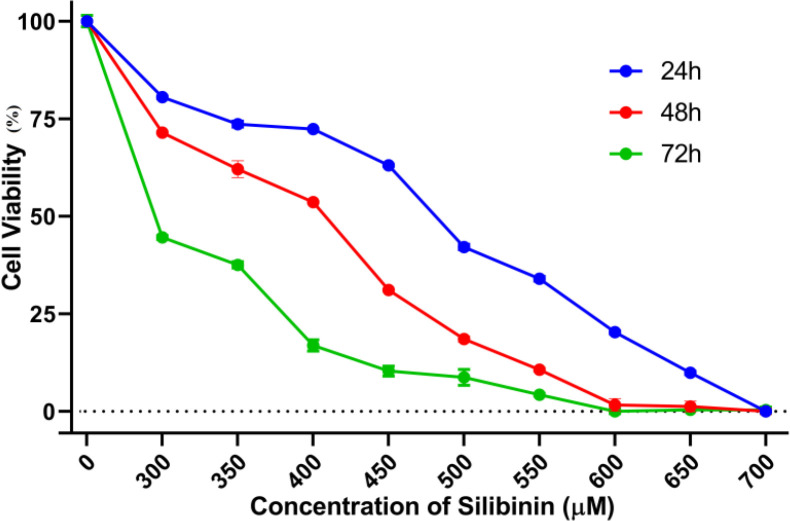
Silibinin inhibited the survival rates of MCF-7 cells. The MCF-7 cells were treated with different concentrations of silibinin (0-700 µM) for 24, 48, and 72 hours, respectively. The findings are presented as mean ± SD (n=3).

**Cell viability investigation:** In order to evaluate how silibinin affects MCF-7 cell survival and proliferation, we conducted an MTT assay, a method that provides a quantitative evaluation of cell viability, allowing for precise measurements of silibinin's effects through the generation of numerical data, thereby facilitating accurate comparisons and statistical analysis. In brief, MCF-7 cells were plated in 96-well plates at a density of 1×10^4^ cells/well and incubated for a full day. After confirming cell adhesion to the plate surface, we added varying doses of silibinin (ranging from 0 to 700 μM) to each well in triplicate. Following this, incubation periods of 24, 48, and 72 hours were implemented (Fig. 1). Subsequently, 5 mg/ml of the MTT reagent in PBS was introduced into each well, and the cells were then subjected to a 37°C incubation for a duration of 4 hours. Aspirating the supernatant and adding DMSO for 30 minutes were performed to facilitate the dissolution of insoluble formazan crystals. As a final step, both samples and control were measured for optical density (OD) using an Elisa microplate reader (RT-2100C Microplate Reader, China) at 570 nm. The determination of the MCF-7 cells survival rate utilized the subsequent formula: (OD of Silibinin-treated cells) / (OD of untreated cells)×100. In this study, the 50% inhibitory concentration (IC_50_) was determined by using GraphPad Prism 8 software.

**Determining Target Genes of miRNA: **The identification of miR-133a targets involved the utilization of multiple online bioinformatics tools. TargetScan, miRDB, miRwalk, miRmap, and miRTarbase are among the algorithms included in this collection. PAM pathway targets were identified as the focus of the analysis (Table 1). MiRNAs and their potential targets are evaluated thermodynamically using these algorithms. By identifying matching binding sites and determining their minimum energy in the 3'-untranslated region (3'-UTR), this evaluation provides insights into the molecular binding mechanism. After cross-referencing results from various databases, only genes anticipated by four or more algorithms were taken into account to mitigate overprediction.

**Table 1:** The target genes of the miR-133a were determined using the target prediction scores from five web applications. The web applications used for prediction included miRDB, miRWalk, TargetScan, miRmap and miRTarBase

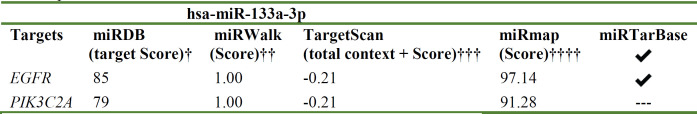

† The higher value of this score indicates a higher likelihood of targeting.

†† The closer this value is to one, the higher the probability that this interaction "works".

††† The higher negative the value of this score, the more effective suppression.

†††† The predicted miRNA repression strengths are displayed as "Percentile" (ranging from 0 to 100%, with 100% representing the greatest repression).

Validation methods with strong evidence (qPCR, Western blot and Reporter assay).

**RNA isolation and qRT-PCR** **:** MCF-7 cells, whether treated or untreated, had their total RNA isolated using RNX-plusTM in accordance with the manufacturer's instructions. The purity and concentration of the isolated RNA were determined using a NanoDrop One UV-Vis spectrometer (Thermo Fisher Scientific, Waltham, Massachusetts) at 260/280 and 260/230 nm. Additionally, the integrity and quality of the isolated RNA samples were determined by performing electrophoresis on 1% agarose gels. Pars Tous Company Kit (Iran, Mashhad) instructions were followed to reverse-transcribe the mRNA into cDNA. The Anacell kit (Anacell; Iran, Tehran) was used according to the instructions provided by the manufacturer to synthesize cDNA specific to miR-133a using a stem-loop primer.

A primer blast with the NCBI primer database was used to evaluate the specificity of primer pairs for mRNAs. The design of the primers was a joint effort between Integrated DNA Technologies (IDT) and Clustal Omega (Table 2). MIMAT0000427 is the accession ID for miR-133a primers. They were developed and compiled by Anacell using miRBase (http://www.mirbase.org). The levels of β-actin and U6 genes were used as internal controls to normalize the expression levels of mRNAs and miRNAs. In this study, qRT-PCR was conducted using the Real Q Plus 2× Master Mix Green kit (Amplicon, Denmark) on the Light Cycler® 96 system (Roche Life Sciences, Deutschland GmbH, Sandhofer, Mannheim, Germany). Both expression mRNAs and miRNA levels were calculated using the 2^-∆∆CT^ approach.

**Table 2:** The primer sequences for qRT-PCR analysis

**Genes**		**Primer sequence (5´→3´)**	**Length of production (bp)**
***EGFR***	Forward	CGCAAAGTGTGTAACGGAATAG	162
	Reverse	CAGAGGAGGAGTATGTGTGAAG	
			
***PIK3C2A***	Forward	TACCAATCACCGCACAAACC	150
	Reverse	ACAGTAGAACTCACATCACACG	
			
***PTEN***	Forward	AGTCCAGAGCCATTTCCATC	183
	Reverse	GATAAATATAGGTCAAGTCTAAGTCG	
			
***AKT-1***	Forward Reverse	ACACCACCTGACCAAGATGA TACAGATCATGGCACGAGG	164
			
***mTOR***	Forward	CAATAGGAGAATTGGCACAGG	206
	Reverse	CAGTAGCACCTCAAGCAAAGT	
			
***β-Actin***	Forward	AATGTGGCCGAGGACTTTG	261
	Reverse	GGCACGAAGGCTCATCATT	

Standard curves have been drawn for all genes before data analysis. Finally, PCR products were visualized with resolution after electrophoretic separation on a 3% agarose gel.

**Western blotting analysis: **For a duration of 48 hours, MCF-7 cells (8×10^5^ cells) were subjected to treatment with silibinin at three concentrations (320, 370, and 420 µM). Subsequently, RIPA buffer was used to lyse the cells, along with phosphatase and proteinase inhibitor cocktails (cOmplete Mini Tablets, PhosSTOP and ProSTOP, Roche, Basel, Switzerland). In order to perform western blot analysis, protein samples were extracted according to the following procedure. In brief, the BCA Protein Assay Kit (DNA biotech; Iran, Tehran) was used to measure protein amounts. An electrotransfer device from Bio-Rad was used to transfer twenty micrograms of protein to nitrocellulose membranes, following loading onto a 4-10% precast polyacrylamide gel according to the instructions provided by the manufacturer. At room temperature, 5% skim milk was applied to the membranes to block them for two hours. Subsequently, they were incubated overnight at 4°C with primary antibodies, including anti-p-mTOR antibody (ab109268, diluted at 1:1000), anti-mTOR antibody (ab134903, diluted at 1:1000), and anti-β-actin antibody (ab115777, diluted at 1:200-500). Following incubation with HRP-conjugated secondary antibodies (1:200-500 dilutions of Ab97051), there was a three-step washing procedure for the membranes with TBS-Tween. Finally, the proteins were visualized using an X-ray film following ECL detection. As far as the rest of the chemicals are concerned, all were purchased from Sigma Chemicals unless specified differently. The band intensities were assessed utilizing Image J software after being normalized with respect to the loading control.

**Statistical Analysis: **A triplicate of each experiment was performed. SPSS 26 software (SPSS Inc., Chicago, IL) and GraphPad Prism 8 software (San Diego, CA, USA) were used for statistical analysis. The one-way ANOVA (analysis of variance) was used to examine differences across groups, and a post hoc test (Tukey) was conducted following the ANOVA. In all samples, statistical significance was attributed to values with a P<0.05, and results are presented as mean ± standard deviation (SD).

## RESULTS

Silibinin exhibited a dose- and time-dependent decrease in the proliferation and viability of MCF-7 cells compared to the untreated control cells (100% cell viability) (Fig. 1). Based on our preliminary investigations, treatment with silibinin (370 μM) resulted in an approximately 50% reduction (IC_50_) in cell viability after 48 hours. We selected the doses of silibinin at 320, 370, and 420 μM for subsequent experiments based on the IC_50_ concentration.

**Figure 2:**
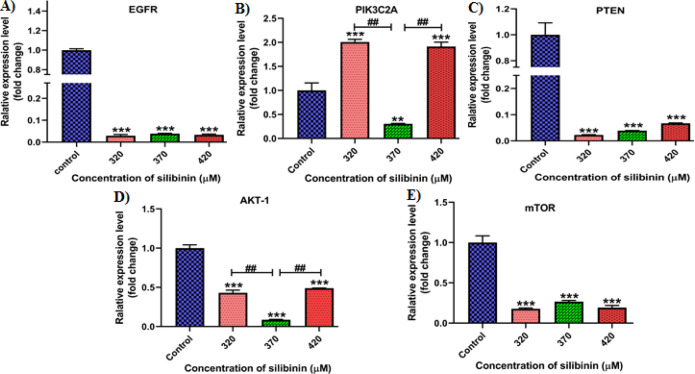
Quantitative expression levels of *EGFR* (A), *PIK3C2A* (B), *PTEN* (C), *AKT-1* (D), and *mTOR* (E) in silibinin-treated cells compared to untreated cells. The results are represented as mean ± SD (n=3). **p<0.01, ***p<0.001, ##Compare with groups p<0.001

The Levels of the mRNAs for *EGFR*, *PIK3C2A*, *PTEN*, *AKT-1*, and *mTOR* in MCF-7 cells were assessed after exposing them to the specified concentrations of silibinin for 48 hours (Fig. 2). The MCF-7 cells treated with 370 μM of silibinin significantly inhibited (p<0.001, Fig. 2A) the expression level of EGFR mRNA, as well as its downstream genes *PIK3C2A*, *PTEN*, *AKT-1*, and *mTOR* (Fig. 2B-E). In contrast, at concentrations of 320 μM and 420 μM of silibinin, there was a significant increase (p<0.001, Fig. 2B) in the expression level of *PI3KC2A*. It appears that while silibinin demonstrates increased cytotoxicity at higher concentrations, the desired biological impact is only attained within specific concentration thresholds (Fig. 2B). These findings indicate that silibinin at a concentration of 370 μM significantly inhibits the transcriptional activity in the PAM signaling pathway. 

**Figure 3:**
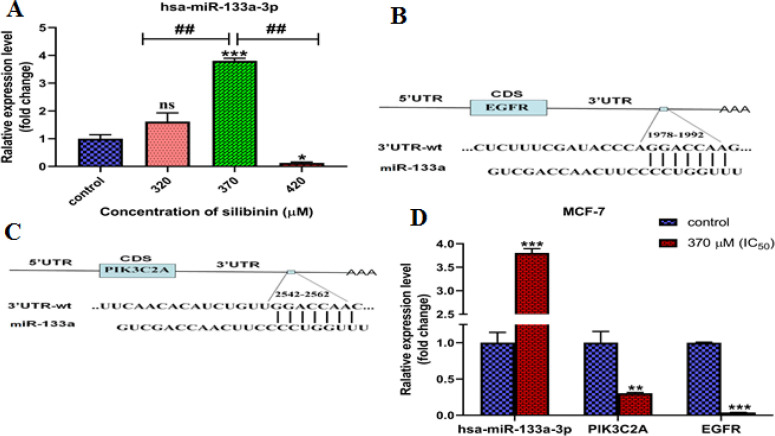
(A) Relative expression levels of miR-133a in silibinin-treated and untreated cells. The relative expression of miR-133a was normalized to U6 small nuclear RNA as an internal control. (B, C) Schematic diagram of the predicted miR-133a binding sites in the *EGFR* 3′UTR and *PIK3C2A* 3′UTR. (D) Comparison of the expression levels of miR-133a, *PIK3C2A*, and *EGFR* genes in cells treated with silibinin (370 µM, IC_50_). The results are represented as mean±SD (n=3). *p<0.05, **p<0.01, ***p<0.001, ##Compare with groups p<0.001

To further enhance our understanding of the action of silibinin against cancer, we analyzed both the level of miR-133a expression in MCF-7 cells treated and untreated after 48 hours. The results revealed a significant increase (p<0.001, Fig. 3A) in the expression of miR-133a upon treatment with 370 μM of silibinin. However, while not reaching statistical significance, there was an observed trend towards increased miR-133a expression at 320 μM. In contrast, miR-133a expression significantly decreased at 420 μM compared to the control group (p<0.05, Fig. 3A).

Bioinformatic analysis suggested that *EGFR* and *PIK3C2A* mRNAs could potentially serve as molecular miR-133a targets in the PAM pathway (Fig. 3B, C). Furthermore, no binding site for miR-133a was detected in the *PTEN*, *AKT-1*, and *mTOR* mRNAs. The correlations between miR-133a and the mRNA expressions levels of *EGFR* and *PIK3C2A* were assessed employing the Spearman's rank correlation test. The findings indicated a significant negative correlation between miR-133a expression and its targets, *EGFR* (r=-0.93, p<0.01) and *PIK3C2A* (r=-0.90, p<0.01). Overall, the increase in miR-133a expression was associated with a decrease in the expression of both *EGFR* and *PIK3C2A* (Fig. 3D).

**Figure 4:**
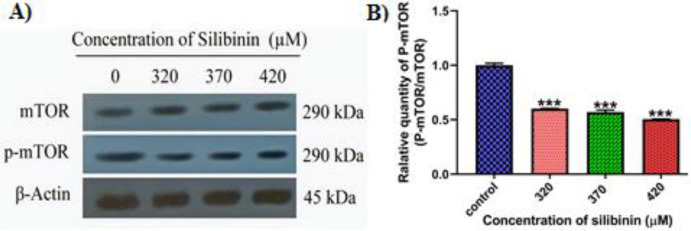
The protein expression levels of mTOR in MCF-7 cells. (A) The effects of various silibinin concentrations (320, 370, and 420 µM) on the relative protein expression of mTOR and p-mTOR in MCF-7 cells were assessed at 48 hours. (B) Immunoblotting analysis of p-mTOR/mTOR. The results are represented as mean ± SD (n=3). *** p<0.001

Consistent with the qRT-PCR findings, western blot analysis unveiled a decrease in the protein levels of p-mTOR (p<0.001), the end product of the PAM signaling pathway, in silibinin-treated cells compared to the control group (Fig. 4).

## DISCUSSION

Breast cancer, globally widespread, affects millions of women each year and shows diverse prognoses despite clinical similarities [14]. Understanding molecular mechanisms is crucial for enhanced patient care [15]. Silibinin and its derivatives exhibit inhibitory effects on various tumors, including breast cancer, in laboratory models [16]. The study presents evidence of silibinin's suppressive impact on breast cancer cells via the PAM pathway, highlighting the regulatory role of miRNAs. These findings reveal promising new avenues for breast cancer treatment.

The PAM pathway alterations are responsible for over 70% of breast cancer cases and contribute to treatment resistance, making it a crucial therapeutic target [17]. *EGFR*, a tyrosine kinase cell surface receptor, regulates multiple downstream effectors such as *PI3K* and *AKT* [14]. *PTEN* acts as a tumor suppressor by diminishing AKT activity via PIP3 reduction. Loss of PTEN function is prevalent in malignancies [18]. *AKT* exists in three isoforms: Among these, AKT-1 is primarily associated with cancer. AKT phosphorylates a variety of substrates, including mTOR, a pivotal regulator of cell growth and metabolism [19] (**Fig. S1**). *PIK3CA*, the most frequently mutated oncogene in luminal breast cancer, can affect the PAM signaling pathway regardless of receptor status, while 4% of cases have mutations of *AKT-1* and *PTEN* [18]. In this study, Cells of the MCF-7 type served as a representative model for luminal breast cancer. Resistance to therapy in this malignancy is influenced through the route of PAM signaling, along with mutations in the PI3K catalytic subunit, activation of *AKT*, and loss of *PTEN* function. Consequently, emerging therapeutic approaches target this pathway with specific medications [20]. 

Our findings indicate that silibinin effectively suppresses the expression of *EGFR* mRNA, a crucial compound in the PAM signaling pathway, along with several downstream genes, including *PI3K*, *AKT-1*, *PTEN*, and *mTOR* mRNAs, in cancer cells (Fig. 2). The inhibitory effect of silibinin results in a reduction in tumor cell growth and development. In this study, our primary focus was on the PAM signaling pathway, which has been previously recognized as significant in breast cancer. In addition, a particular primary antibody against mTOR was employed in western blot analysis to acquire a better understanding of how this pathway's end product interacts with other components of signaling cascades.

The studies indicate that silibinin could be an effective drug for inhibiting EGFR expression in cancer cells. Kim et al. found that silibinin reduced EGFR ligand-induced *CD44* expression in breast cancer cells [21]. In another study, it was revealed that silibinin prevents the advancement of renal cell carcinoma induced by EGFR signaling through the inhibition of the EGFR/MMP-9 pathway [22]. The studies indicate that silibinin's anticancer effects could be attributed to its inhibition of the PAM pathway in cancer cells. Li et al. discovered that silibinin inhibits the PAM pathway in rhabdoid tumor G401 cells, resulting in the prevention of cell migration and invasion [23]. In this current study, we observed that silibinin can exhibit varied effects on *PIK3CA* expression at different inhibitory concentrations (Fig. 2B), aligning with the results of Jahanafrooz et al., who documented diverse inhibitory effects of silibinin in breast cancer cells [24]. On the other hand, as mentioned earlier, PIK3CA can function regardless of the receptor status [18]. Several studies have documented that silibinin increased *PTEN* mRNA expression levels in MCF-7 and MCF-10A cell lines; however, this effect was not observed in the T47-D cell line [24, 25]. Throughout our research, we recognized that silibinin reduced the expression of *PTEN* mRNA in MCF-7 cells. Nevertheless, this result did not align with increased activity in the PAM pathway. Interestingly, another study suggested that silibinin did not impact *PTEN* expression in MCF-7 cells, implying a potential involvement in upregulating *P53* expression within breast cancer cells [26]. It is possible to conclude that silibinin, besides its role as a tumor suppressor, may also decrease the expression of certain tumor suppressor genes or enhance the expression of specific oncogene genes.

As a non-invasive biomarker, miR-133a has a vital function in diagnosis, disease progression, and treatment response. MiR-133a has emerged as a tumor suppressor in non-small cell lung cancer (NSCLC), esophageal cancer (EC), prostate cancer (PC) and breast cancer (BC), targeting genes such as *SOX4*, *EGFR*, *FSCN1*, and *COL1A1* [5]. Evidence suggests that miR-133a has the capability to inhibit the PAM pathway through its targeting of EGFR, thereby positioning it as a promising downstream target for suppressing cell proliferation in breast cancer [27, 28]. MiR-133a exhibits the potential to induce cellular apoptosis and suppress cell proliferation in NSCLC cells, a process facilitated by its specific targeting of the EGFR/AKT/ERK signaling pathway [29]. Moreover, miR-133a was identified as contributing to the promotion of prostate cancer bone metastasis by downregulation, potentially mediated through its targeting of EGFR and inhibition of the PAM signaling pathway [30]. 

Herbal flavonoids such as quercetin, luteolin, curcumin, and silibinin have positive effects on different cancers by regulating miRNAs, indicating the potential of targeting miRNA pathways as a promising approach in cancer treatment [10]. According to Melkizadeh et al., silibinin significantly decreased miR-21 and miR-155 expression in MCF-7 cells. As a result, there was an increase in the expression of Caspase-9, P53, BID, and APAF-1, which are the targets of miR-21 and miR-155 [31]. Yazdi et al. found that both free silibinin and TMC-coated silibinin decreased the expression levels of miR-141, miR-15a, and miR-21 in T47D cells. Consequently, cell survival declined and apoptosis increased. Notably, the TMC-coated silibinin exhibited higher effectiveness compared to the free form [32]. Additionally, it was discovered that luteolin exhibited a synergistic effect with silibinin in glioblastoma multiforme cells (U87MG, T98G), leading to an upregulation of miR-7-1-3p expression [33]. In our study, it was discovered that exposing MCF-7 cells to silibinin enhances the efficacy of miR-133a (Fig. 3A). It appears that miR-133a expression is affected by silibinin in a concentration-dependent manner. This, in turn, allows miR-133a to suppress the PAM signaling pathway in tumor cells by reducing the expression of EGFR and PIK3C2A targets (Fig. 3D), ultimately resulting in decreased growth and development of tumor cells. The effect of silibinin on the PAM pathway can be either miR-133a-dependent or miR-133a-independent. To differentiate the specific impact of silibinin from miR-133a, future investigations should involve knockout and knockdown of the miR-133a gene. This suggests that combining miR-133a and silibinin has the potential to be a complementary treatment for breast cancer, but further clinical studies are needed to support this possibility.

Utilizing flavonoids for cancer treatment poses challenges such as low solubility and targeted delivery of cancer-specific agents [34]. To enhance the efficacy of silibinin, future studies should investigate stable complexes of silibinin and nanoparticles. This approach offers the potential to develop natural substances that specifically target miRNA, introducing a novel and promising strategy for cancer treatment.

While our study has provided valuable insights into the effects of silibinin on MCF-7 cells, several limitations must be taken into account. Using only one cell line, MCF-7, is a notable limitation for our experiments. While this cell line is a widely used model in breast cancer research, it is essential to recognize that the responses of different breast cancer subtypes or other types of cancer cells may vary. To enhance the generalizability of our results and establish the broader relevance of silibinin as a potential therapeutic agent, further studies involving a diverse range of breast cancer cell lines and *in vivo* models are warranted. Such investigations will provide a more comprehensive understanding of silibinin's efficacy and safety, helping to bridge the gap between *in vitro* findings and potential clinical applications.

### Conflict of Interest:

The authors declare that there is no conflicting of interest.

### Authors’ Contribution:

Conception and design of the study, data collection and analysis, and manuscript writing: Mj. Hossein-Tehrani. Manuscript revising and final approval of the manuscript for submission: R. Abbasalipourkabir. Project supervisor: N. Ziamajidi**.**

## Supplementary materials

Figure S1
