# Supplementary material for: The role of miR-133a in silibinin-mediated inhibition of the PI3K/AKT/mTOR pathway in MCF-7 breast carcinoma cells
Source: Mol Biol Res Commun. 2024;13(2):79–83. doi: 10.22099/MBRC.2024.48818.1903 (PMC10946549; doi:10.22099/MBRC.2024.48818.1903)
Supplement: Figure S1 [file mbrc-13-73-s001.pdf]

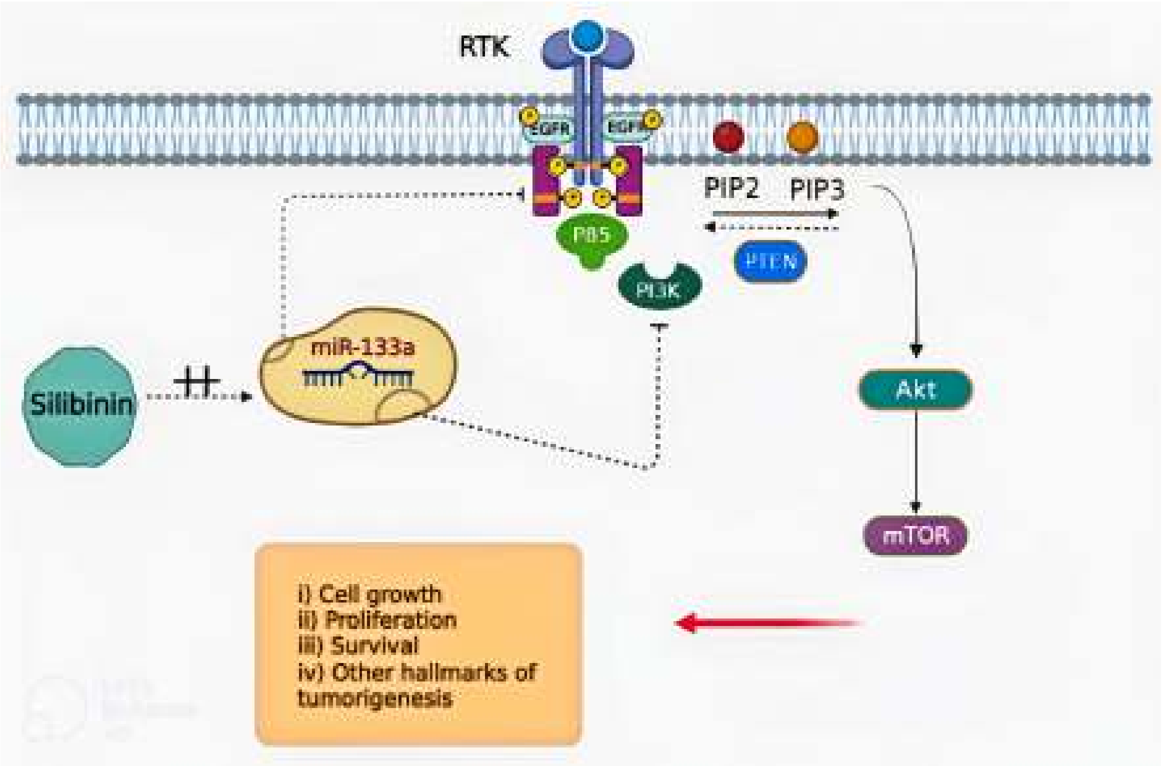

Figure S1: Overview of miR-133a /EGFR/ PI3K signaling pathway. Class I PI3Ks are activated through GPCR or RTK receptors. PTEN functions as a tumor suppressor and inhibits PAM signaling pathway. Herbal flavonoids can regulate miRNAs, leading to the reduction of carcinogenesis, tumor growth, and inhibition of cancer cell malignancy.
